# Impact of Antemortem Handling on the Behaviour of Holstein and Commercial Crossbred Steers and on the Incidence on Dark-Cutting Beef

**DOI:** 10.3390/ani16101457

**Published:** 2026-05-09

**Authors:** Fernanda Alein Chávez-Balderas, Rubén Danilo Méndez Medina, Luigi Faucitano, Francisco Alejandro Ruíz-López, María Salud Rubio Lozano

**Affiliations:** 1 Faculty of Veterinary Medicine and Animal Science, National Autonomous University of Mexico (UNAM), Ciudad Universitaria, Av. Universidad #3000, Coyoacán, Mexico City 04510, Mexico; alein.vet@gmail.com (F.A.C.-B.); menmedanilo50@gmail.com (R.D.M.M.); mvzfrancisco.ruiz@gmail.com (F.A.R.-L.); 2 Agriculture and Agri-Food Canada, Sherbrooke Research and Development Centre, 2000 College Street, Sherbrooke, QC J1M 0C8, Canada; luigi.faucitano@agr.gc.ca; 3 Meat Science Laboratory, Centro de Enseñanza Práctica e Investigación en Producción y Salud Animal (CEPIPSA), Avenida Cruz Blanca No. 486, San Miguel Topilejo, Alcaldía Tlalpan, Mexico City 14500, Mexico

**Keywords:** ante-mortem handling, animal welfare, Holstein, dark cutting

## Abstract

Dark cutting (DC) is a significant quality defect in beef, often linked to stress experienced by animals prior to slaughter. This study evaluated how antemortem handling affects behavior and DC incidence in Holstein (*n* = 101) and commercial crossbred (CC; *n* = 101) steers finished under the same commercial conditions in northwestern Mexico. Animal welfare and handling practices were assessed during driving, transport, lairage, stunning and bleeding, and carcass and meat traits related to DC were measured. Holsteins, originating from confined systems with high human–animal interaction, showed more backing events than CC (73 vs. 30; *p* = 0.03), which led operators to increase vocal encouragement (109 vs. 5; *p* < 0.001) and hitting (100 vs. 28; *p* = 0.002). Additionally, 56.4% of Holstein carcasses had L* ≤ 40 compared with 22.8% in CC (*p* = 0.001), whereas a greater proportion of CC carcasses exhibited ultimate pH ≥ 5.8 (46.5 vs. 20.8%; *p* = 0.03). Meat from Holstein steers showed lower lightness, lower redness and higher yellowness, together with a higher incidence of DC (17.8 vs. 10.9%). In conclusion, genetic background and the human–animal relationship influence responses to antemortem handling and the incidence of dark cutting.

## Simple Summary

In many countries, Holstein males are increasingly finished for beef production, but they may react differently to handling at the farm and slaughterhouse than traditional beef breeds. In this study, we compared Holstein steers with commercial crossbred beef steers under the same commercial feedlot and abattoir conditions in Mexico. We recorded animal behavior and stockperson practices during loading, transport, lairage, driving to the stunning box and slaughter, and then evaluated meat quality, focusing on traits related to dark cutting. Holstein steers, which came from confined dairy systems with frequent human contact, stopped and backed more often during handling and required more vocal encouragement and hitting to move. They also produced darker meat and a higher proportion of dark-cutting carcasses than crossbred beef steers. These findings suggest that genetic background and previous human–animal interactions influence how cattle respond to commercial handling, and that standard beef-handling procedures may need to be adapted for Holstein steers to improve welfare and reduce economic losses from dark cutting.

## 1. Introduction

In the Laguna region and other portions of Mexico, the finishing of male Holsteins has increased to supply domestic and international beef demand. The quality and yield of beef derived from Holstein cattle have been previously studied [1,2,3,4]. A comparative study of beef breeds and Holstein cattle [5] emphasized the dynamic nature of tissue development, which involves sequential changes in muscle, fat, and bone deposition throughout the animal’s productive life, as well as the impact of physiological maturity on final carcass composition, considering that Holstein is a late-maturing breed compared with specialized beef breeds, which are early-maturing. This growth pattern directly influences the muscle-to-fat ratio, marbling distribution, and overall carcass yield, which significantly affect meat quality and commercial grading.

Although animal welfare assessment in Holstein cattle feedlots in Mexico has been studied [6], as well as the relevance of animal welfare and its influence on dark cutting (DC) [7], there is limited information on the handling of Holstein cattle in feedlots, its assessment, and its effect on meat quality. One of the main consequences of poor ante-mortem handling of cattle is so-called DC, which causes major economic losses [8]. According to the Beef Cattle Research Council [9], in 2021, the incidence of DC in youthful fed slaughter in Canada averaged 1.25%, with the resulting discounts exceeding $10 million in the Canadian industry in 2016–2017. In the United States, the Agricultural Research Service [10] documented a national DC incidence of 3% in carcasses in its 2022 annual report, and Riggs et al. [11] reported that Bratzler’s [12] loss estimate of roughly $1 million in 1946 corresponds to an inflation-adjusted value of $20.2 million in 2022 US dollars. In Mexico, there are no official data on the incidence of DC or the economic losses caused by this defect [13]; however, studies in the northeast of the country have reported rates of 13.45% [14].

The economic losses associated with meat with this defect are mainly due to the rejection of its colour by consumers, who seek meat with a bright cherry-red colour and tend to reject any deviation from that criterion because it is associated with lower meat quality [15,16,17].

Önenç [18] documented the incidence of DC in Holstein-Friesian cattle under commercial slaughter conditions, noting that these animals are particularly susceptible to producing darker-coloured meat and other characteristics associated with the defect under commercial handling protocols. Similarly, Ardicli et al. [19] showed that, in male Holsteins, variation in meat colour is directly influenced by elevated post-mortem pH values. Beyond colour, DC meat exhibits abnormalities in other quality traits, such as elevated pH and water-holding capacity [20], reduced shelf life [21] and even sensory differences in flavour [22].

Previous studies have identified causes that can lead to DC in the carcass [8,23] and have concluded that one of the main drivers is the absence or deficiency of animal welfare during the 24 h prior to slaughter [24,25]. Although additional factors such as diet composition, feed withdrawal, transport conditions and environmental conditions may also influence muscle glycogen depletion, ante-mortem handling and the associated stress response remain among the primary determinants of DC occurrence. In fact, pre-slaughter handling (from the production unit to the abattoir) is the primary determinant of DC occurrence, because it is associated with prolonged stress that depletes muscle glycogen reserves, causing the final pH (24 h post-mortem, or ultimate pH [pHu]) to remain above 5.9 [26,27,28].

In this context, it has also been shown that DC incidence is related to animal temperament, because some individuals exhibit more aggressive or fearful responses that make them more difficult to handle, due to prior negative experiences or genetic factors [29,30,31]. Cattle with a more excitable temperament are known to be more prone to stress, and this greater susceptibility to stress affects meat quality [32] through the occurrence of defects such as DC. In addition, Holstein cattle generally originate from housed systems in which animals are exposed to early contact with humans compared with specialized beef cattle, which are raised in extensive systems and have low human interaction until the finishing stage.

Therefore, it is of great importance to determine whether Holstein (HOL) and commercial crossbreds (CC) exhibit differences in behaviour during ante-mortem handling by assessing animal welfare 24 h prior to slaughter, and to identify the impact of animal welfare status on DC incidence. In Mexico, CC cattle typically result from crosses between *Bos taurus* (Bt) European breeds (e.g., Charolais, Simmental, Angus) and *Bos indicus* (Bi) breeds (e.g., Brahman, Indobrasil); Bt cattle have greater muscle mass and fat deposition capacity, while Bi cattle exhibit superior adaptation to tropical and subtropical climates [33].

## 2. Materials and Methods

### 2.1. Animals

This study was conducted under non-invasive observational conditions at a commercial feedlot and abattoir. The research team did not intervene in animal handling or management decisions. Authorization to conduct observations was granted by the facility management, and all commercial procedures complied with NOM-033-SAG/ZOO-2014 [34] and NOM-051-ZOO-1995 [35].

Animal welfare during ante-mortem handling was evaluated in a total of 202 intact male steers younger than 30 months of age (i.e., 24–27 months old at slaughter) at a feedlot in northwestern Mexico. The sample was divided into two genetic groups: (1) 101 Holstein steers (HOL) originating from dairy production systems and born and pre-fattened in the Lagunera region under confined housing conditions until reaching an average body weight of 280 kg; and (2) 101 commercial crossbred (CC) steers intended for beef production, born and raised under extensive systems in southeastern Mexico and later transported to the feedlot with an average weight of 250 kg. Both genetic groups were housed in separate pens according to genotype and underwent a 180-day finishing period. Animals were fed a finishing diet containing 68.8% rolled corn, and both groups received the same total mixed ration throughout the finishing period. Zilpaterol hydrochloride was administered for 30 days, followed by a 5-day withdrawal period prior to shipment to the slaughterhouse. Steers were transported to slaughter at an average live weight of 503 kg.

### 2.2. Assessment of Antemortem Handling and Behavior

The assessment of animal welfare during handling was performed in person on the day of loading by three trained veterinarians and two veterinary interns, following Welfare Quality^®^ (Lelystad, The Netherlands) protocols for beef cattle [36] and the guidelines described in Recommended Animal Handling Guidelines & Audit Guide: A Systematic Approach to Animal Welfare [37] (see Table 1 and Table 2 below).

All assessments were conducted in July, during the summer season in northwestern Mexico. Three loads per genetic group were evaluated on the same day. Feed was withdrawn prior to loading, following the facility’s standard pre-shipment protocol.

The beginning of movement was defined as the moment the first animal in the evaluated group lifted a hoof to move forward, and the end of movement was defined as the moment the last animal ceased forward movement or entered the truck. Data were recorded individually for each steer that exhibited a given indicator, and all assessments were conducted in real time during each loading event.

During loading at the feedlot, personnel followed the company’s daily operational schedule. Movement of animals from the pen to the loading ramp began at 5:10 a.m., 6:23 a.m., and 7:25 a.m. (GMT-5) for the three CC loads, and at 10:18 a.m., 10:37 a.m., and 12:45 p.m. for the three HOL loads. The same three facility employees handled both genetic groups throughout the loading process. The average time from the animals’ exit from the pen to the closure of the truck was 43 min for CC and 50 min for HOL. The loading corridor consisted of solid walls, measured 20.24 m in length and 0.60 m in width, and had a grooved concrete floor with a 2.5° slope. No artificial lighting or light gaps were present in the loading area.

Operational variables were recorded during transport. For CC, driving began between 5:10 a.m. and 7:25 a.m., with feedlot departure occurring between 5:22 a.m. and 7:37 a.m. Three trips were made from the feedlot to the slaughterhouse on the same day, using the same truck and compartments, with an average duration of 16.5 min. Loads consisted of 35, 33, and 33 animals, respectively, with compartment distributions of 16/15/4, 13/16/4, and 15/15/3 animals and an average stocking density of 1.17 m^2^ per animal.

For HOL, driving occurred between 10:18 a.m. and 12:45 p.m., with feedlot departure between 11:36 a.m. and 12:54 p.m. Three trips were also made on the same day, using the same truck and compartments, with an average transport duration of 18.6 min. Loads consisted of 40, 40, and 21 animals, with compartment distributions of 20/16/4, 20/16/4, and 6/15/0 animals, respectively, and an average stocking density of 1.24 m^2^ per animal. Differences in stocking density reflected variations in body size, number of animals per load, and specific operational decisions. The temperature–humidity index (THI) remained within acceptable limits for both genetic groups (67.36 ± 17.90 for CC and 72.01 ± 5.47 for HOL) and was therefore not considered a stress factor under the conditions of this study.

**Figure 1:**
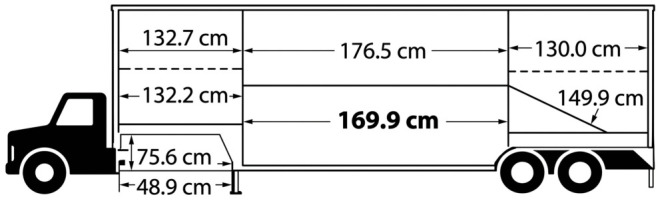
Wilson Silverstar Cattle/Calf Model 402 livestock trailer.

Transport was conducted using a Wilson Silverstar Cattle/Calf Model 402 livestock trailer (Wilson Trailer Company, Sioux City, IA, USA) (Figure 1).

The unloading assessment began at 07:54 a.m. for the CC steers and at 1:30 p.m. for the HOL steers. For CC, the three loads arrived at the slaughter facility at approximately 5:38 a.m., 6:51 a.m., and 7:54 a.m.; for HOL, at approximately 11:52 a.m., 12:14 p.m., and 1:30 p.m. The abattoir where cattle were unloaded has a single unloading point without a ramp. The livestock trailer is directly aligned with the floor of the lairage pen, allowing animals to exit the trailer without changes in elevation. Unloading time was defined as the interval from the moment the first animal’s front limbs exited the truck until the last animal exited the vehicle, with an average of 4 min per load for both genetic groups. Once at the abattoir, animals were distributed into four pens (HOL) and three pens (CC) (Table 3), following the company’s established logistics. Animals remained in these pens for 24 h prior to slaughter, at the corresponding stocking densities. Each pen was equipped with one communal water trough with continuous water supply that adequately met the water requirements of the animals throughout the lairage period.

The human–animal relationship assessment was conducted in the lairage pen of the slaughter facility, four hours after unloading, to minimize the effect of transport stress on animal behavior during the avoidance distance test.

### 2.3. Slaughter Evaluation and Carcass Bruising

The slaughter facility operated under Federal Inspection Type (TIF) conditions, with a daily slaughter capacity of 450 cattle, and complied with all applicable Mexican federal regulations [34,35,39,40,41,42,43]. Both genetic groups were slaughtered on the same day at the same facility to ensure comparable environmental and handling conditions.

Carcass bruising was evaluated according to the criteria described by the Welfare Quality^®^ protocol [36], at the station for meat and hygiene control, between the points where the skin is removed and where trimming occurs. Bruises were classified based on anatomical location (leg, flank, rib, shoulder, anterior back, middle back, posterior back, rump tip, and hip tip) and assessed according to two characteristics: spread—slight (S, 2–8 cm), medium (M, 8–16 cm), and heavy (H, >16 cm)—and depth, where bruises involving tissue beyond the surface muscle were classified as deep (d), resulting in six categories: S (2–8 cm), Sd (2–8 cm, deep), M (8–16 cm), Md (8–16 cm, deep), H (>16 cm), and Hd (>16 cm, deep). Bruises below 2 cm in diameter, fire bruises, and shackling bruises were not recorded. No bruises larger than 25 cm in diameter were observed.

### 2.4. Meat Quality Evaluation

Forty-eight hours post-mortem, carcasses were evaluated under refrigerated storage. Ultimate pH (pHu), instrumental colour, and water-holding capacity (WHC) were assessed in the longissimus dorsi (LD) muscle between the 12th and 13th ribs. Muscle pH was measured directly using a potentiometer with automatic temperature compensation (HI 8521, Hanna Instruments, Inc., Woonsocket, RI, USA) and equipped with a penetration electrode; the potentiometer was calibrated every 10 measurements with pH 4.0 and pH 7.0 buffers [44,45].

Instrumental colour was determined following AMSA recommendations [46] using a HunterLab MiniScan spectrophotometer (Hunter Associates Laboratory, Reston, VA, USA) with a 2.54 cm aperture, illuminant A, and a 10° standard observer, and results were expressed as Commission Internationale de l’Ḗclairage (CIE) L*, a*, and b* values. Each sample was read twice on the ribeye surface after a 15 min blooming period at room temperature, and the mean value was used for statistical analysis.

WHC was measured using the filter paper press (compression) method. Samples were collected directly from the longissimus dorsi muscle of each carcass at the slaughterhouse, immediately after the 15-min blooming period. Briefly, a 1 g meat sample was placed on filter paper, compressed between two polycarbonate plates with a 2.25 kg weight for 5 min, and the areas of the pressed meat and exudate were determined with a digital planimeter (Placom KP-90N, Koizumi Sokki Mfg. Co., Ltd., Nagaoka, Japan); measurements were performed in duplicate. WHC was expressed as the ratio of meat area to exudate area (M/T) × 100.

To determine DC incidence, the frequency of carcasses that showed DC-associated values were compiled and compared by genetic group, based on the following criteria [28,47]: pH ≥ 5.8; L* ≤ 40; a* > 20; and WHC > 50%. DC was determined according to the relationship pH ≥ 5.8 L* ≤ 40.

### 2.5. Statistical Analysis

To compare the results by genetic group for the parameters measured in the animal welfare assessment during ante-mortem handling, the Wilcoxon Mann–Whitney test was used, which allowed us to associate behaviour with handling and the animal’s genetics independently.

Student’s *t*-test was used to compare meat quality between groups, as well as animal welfare measurements recorded during handling at the abattoir, and to determine whether the difference in means between genetic groups was significant. Odds ratios and confidence intervals were also used to determine and compare the relationships between human–animal relationship indicators in the two genetic groups.

The linear correlation coefficient was calculated to measure the relationship between carcass quality variables (dependent) and animal welfare variables in the abattoir (independent). Because no statistically significant difference was found, a principal component analysis was performed to determine whether the dispersion of data differed by genetic group. Finally, to calculate differences in DC incidence, the Wilcoxon Mann–Whitney test was used again. Analyses were conducted using IBM SPSS Statistics 25.0^®^ for Windows (IBM Corp., Armonk, NY, USA). Differences were considered significant when (*p* < 0.05). RStudio® version 1.1.463 (RStudio, Inc., Boston, MA, USA) was used for the principal component analysis.

## 3. Results

### 3.1. Animal Driving

The average driving time from the pen to the loading area and subsequent loading onto the truck was 50 min for HOL and 43 min for CC (Table 4). Vocal encouragement was the most frequently used handling practice, recorded in all three loads for both genetic groups. The number of hits was significantly higher in HOL (100 events) than in CC (28 events; *p* = 0.002). Backing events were significantly more frequent in HOL (73 events) than in CC (30 events; *p* = 0.03).

### 3.2. Lairage Pen (Human–Animal Relationship)

During lairage, a marked difference in human–animal interactions was observed between the two genetic groups. A greater number of HOL steers (44) allowed direct physical contact with handlers, whereas none of the CC steers allowed touching. Additionally, 89 HOL steers remained at distances shorter than 100 cm from personnel, whereas 55 CC steers consistently remained at distances of greater than 100 cm. Statistical analysis showed that HOL steers were 77.19 times more likely to allow human contact than CC steers (Table 5). These findings indicate that HOL steers, reared under confined system conditions with frequent human exposure, developed a closer human–animal relationship, while CC steers, raised in extensive systems, were more reactive and maintained greater avoidance distances.

In this study, finishing conditions were comparable for both genetic groups with respect to human contact, handling practices, feeding, and facilities. However, HOL steers were reared in a confined housing system for 13 months, while CC steers were reared in an extensive production system. Therefore, it may be inferred that these prior human–animal interactions are retained and may continue to influence animal responses during the finishing stage.

### 3.3. Driving from Lairage to the Stunning Box

The average driving time from the lairage pen to the stunning box was 6.5 ± 1.2 min in HOL steers and 5.3 ± 0.9 min in CC steers (Table 6). Environmental noise levels were higher for HOL (72 ± 4 dB) than for CC (68 ± 3 dB); noise was measured continuously throughout the entire handling period using a digital sound level meter (TES 1353™), and the reported value represents the average recorded during the driving stage. Lighting intensity was similar between groups (320 ± 15 lux). The frequency of vocalizations was higher in HOL (28%) than in CC (14%).

### 3.4. Stunning Box

Although no significant difference was observed between groups in the time interval from stunning to bleeding, the recommended time limit was exceeded for a higher proportion of HOL steers (98.8%) than for CC steers (85.1%). This difference was likely due to increased head movements in HOL steers inside the stunning box, which delayed the operator in correctly placing the captive bolt.

In summary, regarding handling practices and driving times, although the same operators and facilities were used for both cattle groups (CC and HOL), the genetic groups’ behavioural responses differed. In both groups, 100% of the animals were successfully stunned with the first shot; however, a greater number of animals in the CC group had correct stun gun positioning compared with the HOL group (11.9 vs. 0). This difference may be associated with greater restlessness in HOL steers, expressed as increased movement in the stunning box, which may have limited appropriate interactions between the animal and the stunning operator.

### 3.5. Bruising

A total of 92% of carcasses presented bruises, with 59% located in the posterior third of the carcass. A higher proportion of HOL carcasses had one or more bruises compared with CC carcasses (88.1% vs. 55.4%; *p* < 0.05), and the total number of bruises was also greater in HOL (176 vs. 75). In HOL carcasses, bruises were mainly located in the mid-dorsal region, whereas in CC carcasses, bruises were predominantly in the anterior dorsal region. These differences were associated with animal behaviour and operator handling during slaughter.

Principal component analysis revealed distinct behavioural patterns in the two genetic groups. In the CC group, the first two components explained 58.7% of total variance and were mainly associated with time in the chute, time in the alley, number of shots, and bleeding time. In the HOL group, the first two components explained 70.8% of variance and were mainly influenced by time in the chute, time in the alley, and noise level, indicating greater sensitivity to environmental stressors.

### 3.6. Principal Components of Abattoir Behavior by Genetic Group

Principal component analysis (PCA) by genetic group was used to identify patterns of association between animal welfare–related variables at the abattoir and the behavioural responses of commercial crossbred (CC) and Holstein (HOL) steers. The variables included animal position at stunning, alley time, noise level, time spent in the stunning box, duration of the tonic phase, pupillary reflex, rhythmic breathing, time between stunning and bleeding, bleeding time, bleed rail sensibility, and the total number of bruises per carcass.

**Figure 2:**
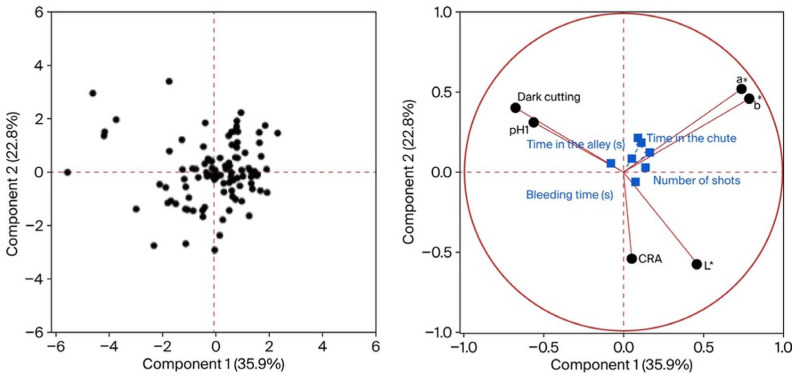
Principal component analysis of animal welfare and meat quality variables in commercial crossbred (CC) cattle. L* = lightness; a* = redness (+)/greenness (−); b* = yellowness (+)/blueness (−); CRA = color rendering ability.

In the CC group (Figure 2), the first two principal components accounted for 58.7% of the total variance. The variables with the highest loading in this group were time spent in the stunning box, alley time, number of shots, and bleeding time. This suggests that the stress experienced in the pre-stunning holding period and operational efficiency—reflected by the number of shots required and bleeding time—may be key factors influencing the responses of animals in this group.

**Figure 3:**
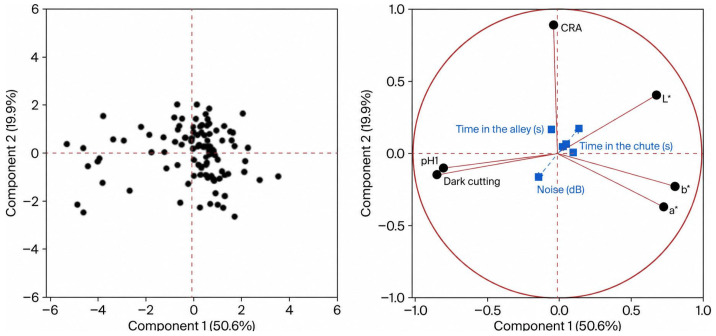
Principal component analysis of animal welfare and meat quality variables in Holstein (HOL) cattle. L* = lightness; a* = redness (+)/greenness (−); b* = yellowness (+)/blueness (−); CRA = color rendering ability.

In the HOL group (Figure 3), the first two principal components explained 70.8% of the total variance, with time spent in the stunning box, alley time, and noise level showing the highest contributions. This indicates that the physical and acoustic environment during ante-mortem handling may have a more pronounced effect in this genetic group, which could be related to a greater susceptibility to environmental stressors.

**Figure 4:**
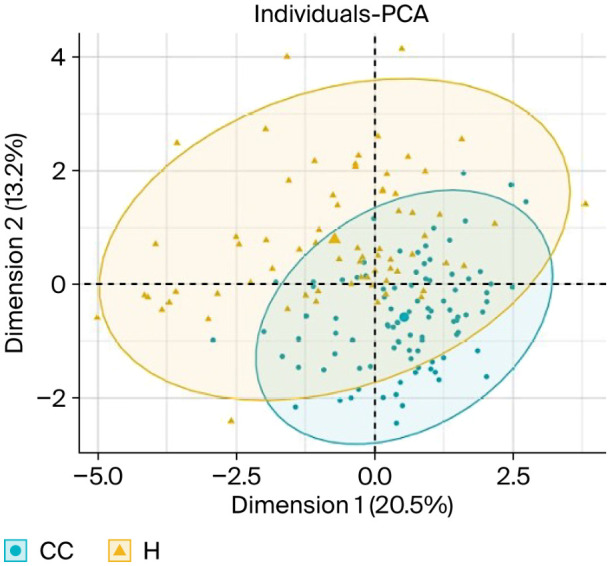
Differences between Holstein (HOL) and commercial crossbred (CC) cattle in the distribution of animal welfare variables at the abattoir.

Finally, the pooled results of the principal component analyses (Figure 4) illustrate the distribution of individuals from both genetic groups across the main axes. The values for the HOL group exhibited greater dispersion, which may indicate higher individual variability in the responses by these cattle to abattoir handling conditions, whereas the values for the CC group appeared more clustered, suggesting more homogeneous behavioural responses. These differences could be influenced by genetic and production characteristics specific to each group, as well as by their degree of prior interaction with humans during handling.

### 3.7. Meat Quality Traits

The evaluation of meat quality revealed significant differences between the genetic groups. The CC group had higher pH values in the longissimus dorsi muscle than the HOL group (*p* < 0.01). When the proportion of carcasses with pH values greater than 5.8 was analyzed, it was significantly higher in the CC group (*p* = 0.03). With respect to colour, meat from HOL steers was darker (lower L* values), yellower (higher b* values; *p* < 0.01), and less red (lower a* values; *p* = 0.02) than meat from CC steers (Table 7). The HOL group had a significantly higher incidence of carcasses exhibiting DC characteristics (*p* = 0.001). Specifically, the frequency of carcasses classified as DC, based on pH48 and L* values, was 17.8% in HOL steers, compared with 10.9% in CC steers (Table 8). However, linear regression analysis using pH, L*, and a* parameters did not reveal a statistically significant relationship among these variables (R^2^ < 0.18), suggesting that the incidence of DC observed in this study may involve additional factors related to handling practices and genetic characteristics.

## 4. Discussion

Notable differences were found in the behaviour of the two genetic groups during ante-mortem handling. During loading and driving, HOL steers required more frequent vocal encouragement and hitting by handlers than CC steers (Table 4). This finding aligns with reports suggesting that temperament and genetic background significantly influence cattle responses to handling [37,48,49,50,51]. In contrast to the study by Blumetto et al. (2017) [52], in which HOL cattle exhibited more agonistic behaviours, the main challenge observed in the present study was their slower response to driving, which may have increased the need for human intervention. The frequent use of electric prods in HOL steers (101 events for *n* = 101 animals) suggests that at least one application per animal occurred, which is a welfare concern that warrants attention in future handling protocols. Together, these findings support the notion that early-life experience and the rearing system (confined housing in HOL vs. extensive systems in CC) may condition animal responses during handling [53,54,55]. Importantly, the loading phase represents a critical welfare risk point, particularly for HOL steers, given the higher frequency of aversive handling events recorded compared to the slaughter phase. The higher number of slips observed in HOL steers during loading at the farm may be related to floor conditions and lower familiarity with handling procedures, whereas the absence of slips during unloading at the abattoir may reflect the level-surface design of the unloading point, which eliminated changes in elevation.

In the lairage pen, HOL steers demonstrated a more positive human–animal relationship, with a higher proportion allowing physical contact compared to CC steers (Table 5). This is consistent with the findings of Silva et al. (2017) [56], who demonstrated that frequent contact with humans promotes trust and facilitates handling. Confined rearing may have favoured positive interactions in HOL steers, whereas CC steers, raised under extensive systems, maintained a greater avoidance distance. These findings are consistent with studies indicating that the human–animal relationship influences both welfare and productivity [57,58,59].

During the movement toward the stunning box, driving times exceeded those considered adequate (<30 min) to reduce the risk of DC [47]. However, differences between genetic groups appeared to be more closely associated with visual distractors and light contrast than with driving time itself. Lima et al. (2018) [60] reported that the removal of such factors reduces cortisol levels, supporting the importance of environmental elements in stress responses.

Environmental noise levels exceeded 85 dB, which is above the tolerable limits [37,61,62,63]. In HOL steers, the primary source of noise was animal vocalizations, whereas in CC steers it was associated with door impacts and vocal encouragement (Table 7). Excessive noise has been shown to increase heart rate and induce fear responses [64,65], which may explain the differences observed between groups.

In the stunning box, although stunning efficacy was 100% at the first shot, HOL steers exhibited greater head movements, which delayed the procedure and increased the stunning-to-bleeding interval beyond recommended limits (<60 s) [66,67]. This was associated with longer bleeding times in HOL steers (Table 6), consistent with the findings in Carrasco-García et al. (2020) [28].

HOL carcasses had a greater number of bruises, which were primarily located in the mid-dorsal region, than CC carcasses, where bruises were more frequently observed in the anterior dorsal region. These results are consistent with Cruz-Monterrosa et al. (2017) [68], who associated bruising with a higher pH and an increased risk of DC, and with Miranda-de la Lama et al. (2012) [69], who reported that more than 90% of carcasses in Mexico have bruises.

Principal component analysis indicated that, in CC steers, stress-related responses were predominantly associated with alley time and bleeding time, whereas in HOL steers, they were more strongly associated with noise levels and time spent in the stunning box (Figure 1, Figure 2 and Figure 3). The greater dispersion observed in the values for HOL steers reflects higher individual variability, as also reported by Blumetto et al. (2017) [52]. The association between alley time and bruising in CC steers may reflect the cumulative effect of animal movement through handling facilities, which increases the likelihood of contact with structural elements at the anterior dorsal region. In HOL steers, the relationship between noise level, time in the stunning box, and bruising suggests that agitation-induced movements within the box may contribute to mid-dorsal injuries.

Regarding meat quality parameters, the CC group had a higher proportion of carcasses with a pH of 5.8 or greater, whereas HOL carcasses exhibited darker meat (Table 8). Elevated pH reflects glycogen depletion associated with stress [70,71,72,73] while darker meat colour has been linked to genetic background and muscle pigmentation [72]. The incidence of dark-cutting carcasses was higher in the HOL group (17.8%) than the CC group (10.9%), with these values similar to those reported by Pérez-Linares et al. (2013) [47] but lower than values reported in commercial populations [74].

These results suggest that the interaction among genetic background, prior human experience, and ante-mortem handling may determine final meat quality, highlighting the need for future research evaluating targeted handling strategies for HOL steers, such as habituation programs and environmental modifications in lairage and stunning areas, to reduce stress-related losses and dark-cutting incidence in commercial operations.

## 5. Conclusions

Genetic background and the rearing system were associated with differences in cattle behaviour, human–animal relationships, and responses to ante-mortem handling in HOL and CC steers. HOL steers exhibited greater familiarity with humans but slower movement during handling, which was associated with increased operator intervention—including higher use of electric prods and hitting—longer stunning-to-bleeding intervals, and a higher incidence of bruising. In contrast, CC steers exhibited more reactive behaviour but were easier to mobilize. These differences highlight the role of handler practices and facility characteristics—including noise levels, floor conditions, and stunning box design—as key factors influencing animal behaviour and welfare outcomes at slaughter.

In terms of meat quality, a higher proportion of CC carcasses had elevated pH, while HOL carcasses tended to be darker and less red and had a higher incidence of dark-cutting characteristics. Overall, these results suggest that genetic background, management history, and ante-mortem handling conditions interact to influence animal welfare at slaughter and the occurrence of dark-cutting beef.

Future research should explore genetic markers for stress susceptibility in HOL steers and evaluate alternative ante-mortem handling protocols—including handler training programs and facility modifications—aimed at reducing dark-cutting incidence in commercial operations.

## Figures and Tables

**Table 1:** Animal welfare assessment criteria [37,38].

Welfare Criterion	Description
Flight zone	During cattle driving, an imaginary line was drawn at the animal’s shoulder to form a 90° angle with the evaluator’s body to move cattle forward, a 45° angle to induce backing, and a 60° angle to stop movement.
Use of electric prods	The evaluator recorded the number of times the operator used an electric prod on any part of the animal’s body during driving.
Facility distractions	Any object or artifact placed along handling alleys, either on the floor or walls, was classified as a distractor. This included lighting failures, light reflections in water puddles, drains, or metal plates, as well as ventilation outlets directing air currents toward advancing animals
Noise	Noise level was measured using a digital sound level meter (TES-1353, TES Electrical Electronic Corp., Taipei, Taiwan); measurements were recorded in decibels (dB), and the source of noise was identified.
Alley time	A standard digital stopwatch (Casio HS-3, Casio Computer Co., Ltd., Tokyo, Japan) with a resolution of 0.01 s and capacity for up to 24 h of continuous measurement was used to record the total time per animal in the alley.
Stunning–bleeding interval	Time elapsed from the onset of the tonic or clonic phase until the carotid and jugular vessels were severed.
Bleeding time	Time elapsed after severing the carotid and jugular vessels, from the onset of blood flow until dripping began.

**Table 2:** Indicators of human–animal relationship and ease of movement [36,38].

Welfare Criterion	Description
Human–animal relationship	Flight zone distance was used to assess the human–animal relationship. Evaluators stood one meter from the animals and performed hand movements to ensure the cattle were aware of their presence in the lairage pen of the slaughter facility. An approach was initiated with the arm over the animal’s head at an approximate 45° angle to the body, with the back of the other hand facing the animal, avoiding direct eye contact. With the evaluator’s gaze directed toward the animal’s muzzle, the evaluator walked toward the animal until withdrawal behavior was observed or until the animal’s nose could be touched. The scoring system was as follows: 0—The evaluator can touch the animal 1—The evaluator can approach closer than 50 cm but cannot touch the animal 2—The evaluator can approach within 100–50 cm; 3—The evaluator cannot approach within 100 cm.
Ease of movement:	Indicators recorded during driving at loading and unloading, at the feedlot, and at the slaughter facility: Number of hits during driving Number of vocal encouragements during driving Number of whistles during driving Number of slips during driving Number of falls during driving Number of backing events during driving Number of turns during driving Number of refusals during driving Number of vocalizations during driving

**Table 3:** Distribution of steers in the abattoir lairage pens.

**Holstein**				
Pen number	1	2	3	4
Number of animals per pen	37	13	20	31
Stocking density (m^2^/animal)	3.04	8.52	5.62	3.54
**Commercial crossbred**				
Pen number	1	2	3	
Number of animals per pen	21	40	40	
Stocking density (m^2^/animal)	5.40	2.79	2.79	

**Table 4:** Evaluation of handler practices and behavioral responses of commercial crossbred (CC; *n* = 101) and Holstein (HOL; *n* = 101) steers during loading and driving at the farm and unloading and driving at the abattoir.

	CC	HOL	
Variables	Number of Events	Number of Events	*p-Value*
**Handler Practices**	**Loading at the Farm**		
Use of electric prod	46	101	0.08
Hits	28	100	0.002
Vocal encouragement	5	109	<0.001
Whistling	4	13	0.32
**Animal responses**	**Loading at the farm**		
Slips	13	41	0.80
Falls	1	2	0.64
Backing events	30	73	0.03
Turns	27	16	0.13
Refusals	33	67	0.42
Vocalizations	17	21	0.75
**Handler practices**	**Unloading at the abattoir**		
Vocal encouragement	2	1	0.68
**Animal responses**	**Unloading at the abattoir**		
Slips	6	0	0.02
Falls	1	0	0.39
Backing events	0	2	0.07
Refusals	0	1	0.25
Vocalizations	2	7	0.66
**Handler practices**	**Driving at the abattoir**		
Vocal encouragement	12	10	1.00
Whistling	20	44	0.21
**Animal responses**	**Driving at the abattoir**		
Slips	1	4	0.25
Refusals	1	2	0.68
Vocalizations	0	1	0.72

Values represent the total number of events recorded across three loads per genetic group.

**Table 5:** Probability of types of human–animal interactions in the lairage pen in commercial crossbred (CC; *n* = 101) steers compared to Holstein (HOL; *n* = 101) steers.

	CC	HOL		
Variables	Odds Ratio	Confidence Interval	χ^2^	*p*-Value
Allows touching	77.19	10.36–567.35	52.87	<0.001
Moves away at 50 cm	19.2	1.45–18.16	16.8	<0.001
Moves away at 100 cm	0.36	0.19–0.68	10.36	<0.001
Moves away > 100 cm	0.11	0.60–0.23	41.29	<0.001

**Table 6:** Noise level and handling time intervals in the stunning alley, stunning box, and bleeding area for commercial crossbred (CC; *n* = 101) and Holstein (HOL; *n* = 101) steers.

	CC		HOL		
Variable	Mean	SD	Mean	SD	*p*-Value
**Handling conditions**					
Noise (dB)	100	±6.47	101	±6.46	<0.001
**Handler practices**					
Alley time (min)	1.58	±0.00	2.17	±0.00	0.26
Stunning–bleeding Interval (min)	1.19	±0.02	2.32	±5.00	0.27
Bleeding time (min)	1.12	±0.01	1.38	±0.02	<0.001

SD: standard deviation.

**Table 7:** Meat quality parameters in commercial crossbred (CC; *n* = 101) and Holstein (HOL; *n* = 101) steers.

	CC		HOL		
Variables	Mean	SD	Mean	SD	*p*-Value
pH48	5.80	±0.12	5.72	±6.72	<0.01
L*	42.29	±3.46	38.89	±4.9	<0.01
a*	26.80	±2.47	25.9	±3.13	0.02
b*	19.04	±2.30	2.60	±2.34	<0.01
WHC, %	42.72	±16.08	39.27	±17.30	0.15

WHC: water-holding capacity.

**Table 8:** Distribution of dark-cutting carcasses in commercial crossbred (CC; *n* = 101) and Holstein (HOL; *n* = 101) steers according to pH_48_, color (L and a), and water-holding capacity (WHC).

	Commercial Crossbred	Holstein	*p*-Value
Carcass Range	%	%	
pH ≥ 5.8	46.5	20.8	0.03
L* ≤ 40	22.8	56.4	0.001
a* > 20	96.0	96.0	0.80
WHC > 50%	37.6	30.7	0.37
pH ≥ 5.8 L* ≤ 40	10. 9	17.8	-
pH ≥ 5.8 a* > 20	1.0	3.0	-
pH ≥ 5.8 WHC > 50%	18.8	5.9	-

## Data Availability

The data presented in this study are available from the corresponding author on reasonable request.

## Abbreviations

The following abbreviations are used in this manuscript:

CC	Commercial crossbred
HOL	Holstein
DC	Dark cutting
SD	Standard deviation
pHu	Ultimate pH
LD	Longissimus dorsi
WHC	Water-holding capacity
